# Influence of Technical Parameters of Carbonization on the Physical and Chemical Characteristics of Materials Obtained by Carbonization of Sunflower Husks from the East Kazakhstan Region

**DOI:** 10.3390/polym18161967

**Published:** 2026-08-12

**Authors:** Aigerim Kaiaidarova, Valeryia Bobrova, Andrei Kasperovich, Sergey Lezhnev, Evgeniy Panin, Sergey Nechipurenko, Sergey Efremov

**Affiliations:** 1 Center of Physical-Chemical Methods of Research and Analysis, Farabi University, Almaty 050040, Kazakhstan; aigerim_ko@list.ru (A.K.); sergey.nechipurenko@kaznu.edu.kz (S.N.); efremsa@mail.ru (S.E.); 2 Department of Polymer Composite Materials, Faculty of Technology of Organic Substances, Belarusian State Technological University, 220006 Minsk, Belarus; bobrova@belstu.by (V.B.); andkasp@belstu.by (A.K.); 3 Graduate School of Metallurgy and Mining Engineering, Rudny Industrial University, Rudny 111500, Kazakhstan; sergey_legnev@mail.ru; 4 Department of Metallurgy and Material Science, Karaganda Industrial University, Temirtau 101400, Kazakhstan

**Keywords:** waste of vegetable raw materials, sunflower husk, char, carbonization temperature, specific surface area

## Abstract

In 2025, the oil and fat industry of the Republic of Kazakhstan showed steady growth, strengthening the country’s position as a major producer and exporter of vegetable oils. However, the production process generates large amounts of waste (up to 100 tons per day), and its recycling is an important part of the oil and fat industry’s economy. High-temperature processing of plant waste has proven to be a promising method for creating new materials for various industries. The aim of this study was to determine the influence of various technical parameters of carbonization (processing temperature and process environment) on the physical and chemical characteristics of materials obtained by carbonizing sunflower seed husks from the East Kazakhstan region at temperatures of 300, 400, 500, 600, 700 and 800 °C in an inert argon environment, as well as by processing the husks in an oxidizing environment at a temperature of 650 °C, for further use in elastomer compositions as new ingredients. Increasing the carbonization temperature in an inert environment led to an increase in the amorphous carbon content, surface porosity, and pH of the studied materials. Another parameter that showed a tendency to increase with increasing temperature in an inert environment was the BET specific surface area. In the case of using an oxidizing environment, the highest pH value was observed, and the formation of crystalline mineral phases was also observed. The differences in the phase states of the studied materials may play an important role in shaping the spatial stack of the polymer matrix when used in rubber compound formulations.

## 1. Introduction

Functional additives are components that are added to materials to improve their properties and characteristics. They are used in small quantities but play an important role in preventing metal corrosion, stabilizing polymers, improving and preserving food products [1], and optimizing the properties of plastics, rubber, paints, printing products, and lubricants [2,3,4]. Functional additives can improve the machinability, performance, appearance, and durability of materials, as well as eliminate or minimize undesirable properties [5]. Although the polymer industry is a major consumer of functional additives, they are also widely used in other fields. Functional additives have a significant impact on the performance and stability of materials, even at low concentrations [6]. The additive market is constantly evolving, offering more complex and multifunctional solutions [7].

Innovations in functional additives for elastomeric compositions include the use of renewable and environmentally friendly resources. Natural oils, bio-fillers, and biodegradable reinforcement materials are viable alternatives to petroleum-based functional additives, reducing the use of fossil resources and environmental impact [8,9,10,11,12]. In addition to improving performance, these bio-additives reduce greenhouse gas emissions throughout the product’s lifetime [13,14,15]. Furthermore, innovations in functional additives reduce the carbon footprint of rubber production and usage. This requires improved invention and production of functional additives for lighter, more cost-effective, durable, and recyclable products. Innovative additives enhance energy efficiency and reduce material waste, making the rubber industry more sustainable and competitive in a rapidly changing global market [16,17]. With the growing demands of the rubber industry, the development and implementation of new technological functional additives for rubber blends based on available plant and mineral resources is crucial.

In recent years, there has been an increasing interest among researchers in the use of carbon-containing materials derived from biomass as new functional additives or new fillers for polymers, as well as for elastomeric compositions, in order to reduce the cost and improve the environmental friendliness of finished products, as well as to modify their strength and performance properties [18,19,20,21,22,23]. The comprehensive processing of plant waste into functional additives or fillers for polymer and elastomeric composite materials is one of the key areas of research in green chemistry and resource conservation. Sunflower husk waste is generated in large quantities during the production of vegetable oil and has a high carbon content and moderate ash content, which makes it a promising material for thermal processing in order to obtain carbon materials with desired properties [24]. The carbonization of sunflower husk under various controlled conditions allows for the production of materials with different physical and chemical surface characteristics, which depend on the nature of the raw material and the parameters of its thermal processing, such as temperature, heating rate, and gas environment (inert or oxidizing) [24,25,26,27,28,29,30,31,32]. A significant number of studies have focused on the characteristics of products derived from sunflower husk and other components, such as their use as sorbents, fuels, and catalyst carriers [25,28,33,34,35,36,37,38]. Additionally, research is actively conducted on polymer composites based on thermosetting and thermoplastic matrices that contain untreated sunflower husk, carbonized sunflower husk, and its derivatives. In the work by Zakrzewska et al. [39], a method for using sunflower husk ash as a filler was proposed, which has a positive effect on the properties of rigid polyurethane coatings (increasing dimensional stability and eliminating the risk of material shrinkage), while in the work by Barczewski et al. [40], it was found that the use of untreated sunflower husk as a filler for the production of low-cost epoxy composites resulted in a decrease in tensile and flexural strength due to the formation of pores and the release of fatty residues from the filler during the exothermic cross-linking process, which led to additional plasticization of the composite. The use of sunflower husk before and after thermal processing in elastomeric composite materials has not been extensively studied.

From the point of view of the materials science of elastomeric compositions, the sunflower husk char obtained under controlled (manipulated) carbonization modes can be considered for use in compositions as a filler due to its high carbon content, or as a functional additive that affects the kinetic parameters of rubber mixture processing due to its high metal impurity content. Changes in temperature and oxygen access conditions can be varied by the physical and chemical characteristics of the char [41,42,43,44,45,46,47,48]:

– Specific surface area and porosity are the main properties that determine the reinforcement efficiency and the possibility of physical adsorption of various low-molecular-weight components of the rubber mixture [25,36,49,50];
– The average size of particles or their aggregates, which affect the rheological characteristics of rubber mixtures and the interparticle interactions in the filler–filler system in the elastomeric matrix [35];
– The chemical composition, as well as the presence of functional groups on the surface, which determine the polarity, wettability, and adhesion to the polymer matrix [39,51,52].

Currently, there are already a number of studies on the physical and chemical characteristics of plant raw materials after temperature treatment, but these studies only cover a limited range of temperatures [53,54], and there is a lack of comparison of the properties of pyrolysis products obtained in different environments. Additionally, research on determining the optimal conditions for producing materials from various types of raw materials is still necessary to identify the optimal processing parameters that would allow the production of char for specific applications. Therefore, there is a need for a targeted study of the physical and chemical characteristics of the char of sunflower seed husks (from the East Kazakhstan region) obtained at carbonization temperatures ranging from 300 to 800 °C in increments of 100 °C in an inert argon environment, as well as a comparison study of the char of sunflower seed husks obtained in an oxidative environment at a temperature of 650 °C, for further use of these materials as fillers and/or functional additives in elastomeric composite materials for various applications.

Unlike most previous studies, which have mainly focused on optimizing char production at one or two selected temperatures for a specific application, this work was designed to study the continuous evolution of sunflower husk during thermochemical conversion over a wide temperature range (300–800 °C) under identical processing conditions. This experimental approach minimizes the influence of secondary process variables and allows for direct evaluation of temperature-induced transformations in the carbon matrix, mineral phases, and surface functional groups. Consequently, the resulting data set provides a comprehensive picture of the gradual transition from partially carbonized biomass to highly carbonized material.

Another distinctive feature of this study is the inclusion of an oxidized ash sample in the same analytical scheme. Although carbonized biomass and biomass ash are typically studied as separate materials, a direct comparison of their structural and chemical characteristics derived from the same precursor through controlled thermal processing remains a rare occurrence. This approach allows us to differentiate between transformations related to the evolution of the carbon matrix and those resulting from the complete oxidation of the organic fraction, providing a deeper understanding of the redistribution of inorganic components and the subsequent transformation of mineral phases during thermal conversion.

Thus, the novelty of this study lies in establishing a systematic relationship between structure and temperature over a wide range of carbonization and directly comparing the carbonization products in an oxygen-free environment with the corresponding oxidized residue obtained from the same biomass. This comprehensive approach provides fundamental information on the thermal evolution of both the organic and inorganic components of sunflower husk, thereby expanding the existing understanding of lignocellulosic biomass conversion and providing a more comprehensive basis for the rational selection of carbonization conditions based on the intended application.

This work, which covers an experimental range up to 800 °C, explores the phenomena of carbon restructuring in the late stages, which are often ignored but are critically important for applications in adsorption, catalysis, and energy storage. Finally, recent studies have confirmed that combining temperature changes with controlled atmosphere selection is essential for purposefully modifying the functional properties of char, but systematic experimental datasets that combine a wide range of temperatures and consistent heating conditions are still limited [55].

## 2. Materials and Methods

**Figure 1:**
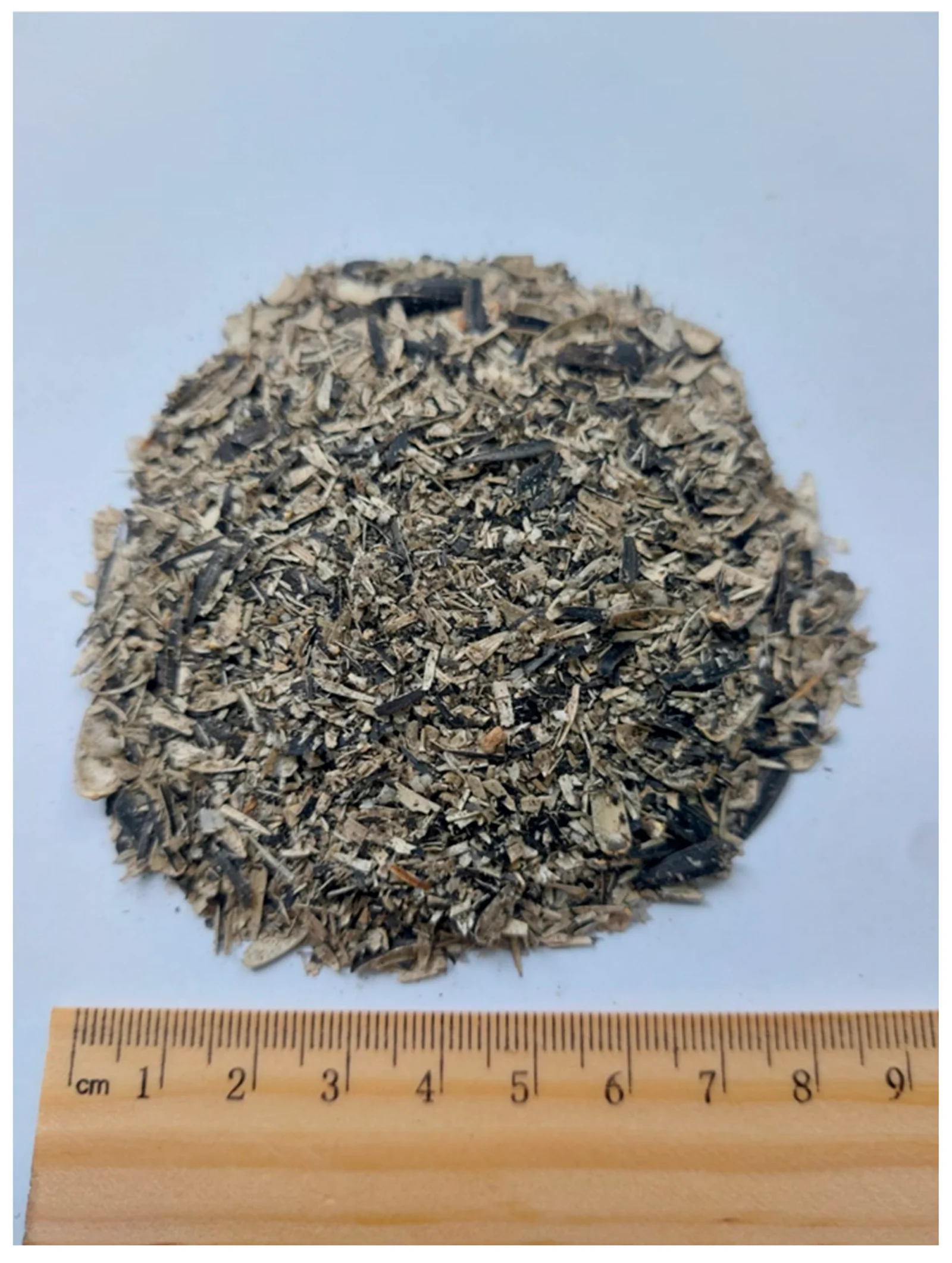
Sunflower seed husks.

The initial raw material for the production of chars was biodegradable waste of plant origin, namely sunflower seed husks (Figure 1), a by-product obtained during the processing of sunflower seeds into sunflower oil. Sunflower is grown in the foothill regions of the East Kazakhstan region, with a total area of more than 5000 hectares. Sunflower (*Helianthus annuus* L.) is the leading oilseed crop in Kazakhstan, with a yield of more than 12% of the total mass.

**Figure 2:**
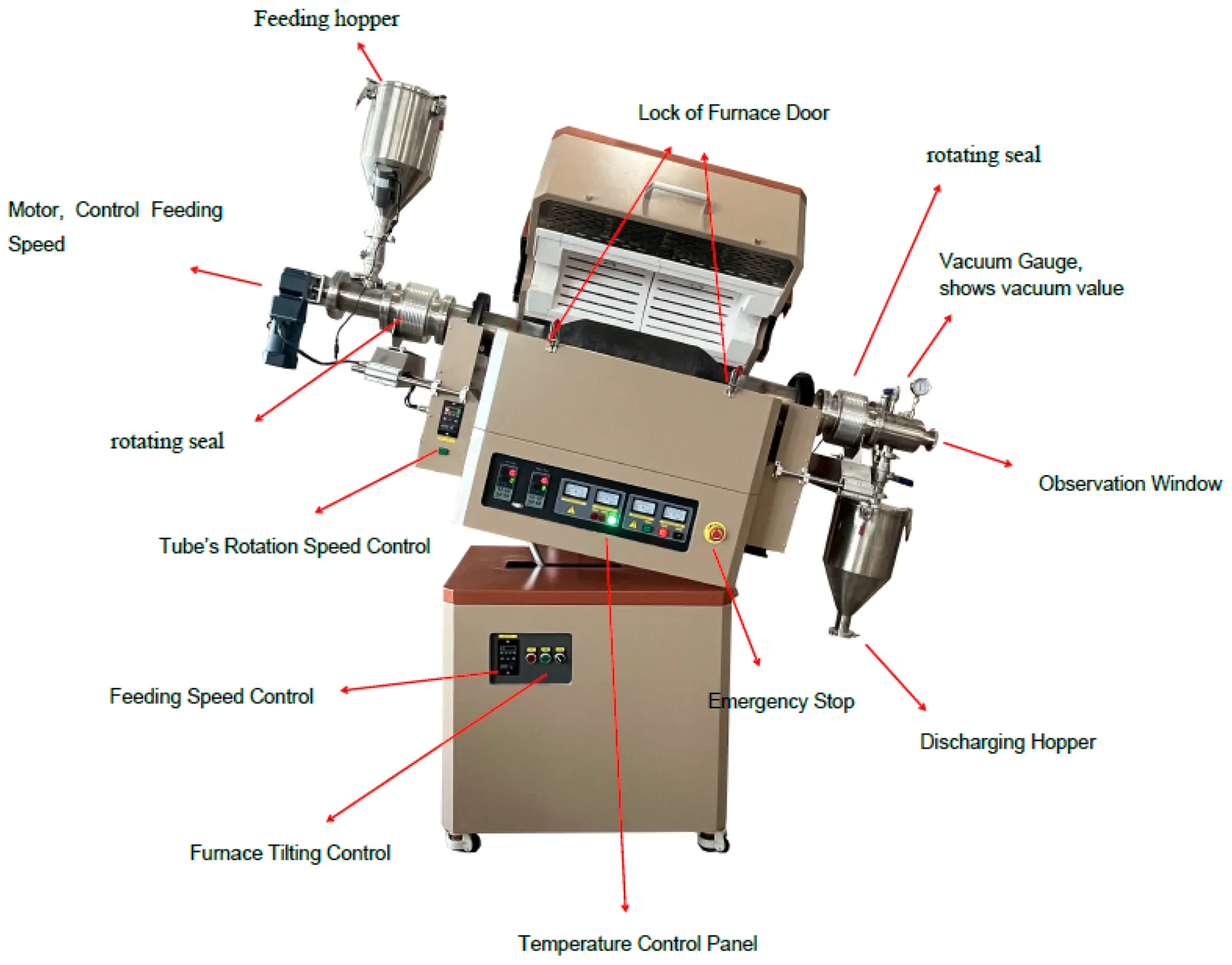
Tubular furnace GSL 1100 XL.

Sunflower seed husks were pyrolyzed in an inclined rotating tubular furnace with a GSL 1100 XL feed and discharge device (Henan Synthe Corporation, Zhengzhou, China) (Figure 2). The husks were loaded into a 5 L receiving hopper and fed through a 50-mm-diameter feeding screw into a rotating retort at a speed of 15 RPM. The retort was flow-through, with a rotation speed of 5 RPM and an angle of 18°. The retort had a volume of 12 L. The carbonized product was discharged from the retort by gravity due to the angle of the retort into a sealed collection hopper. In the hopper, the carbonized product was cooled to ambient temperature.

To study the changes in the physical and chemical characteristics of sunflower seed husks, several processing modes were used:

– Carbonization in an argon-filled furnace (1 dm^3^/min) at a temperature of 300 to 800 °C in increments of 100 °C. The retort was rotated at a speed of 5 RPM. The material was kept in the carbonization zone for 50 min. The resulting chars had specific codes depending on the production temperature (SHC_300_, SHC_400_, SHC_500_, SHC_600_, SHC_700_, SHC_800_, where SHC—sunflower husk char);
– Carbonization in a furnace in an oxygen environment at a temperature of 650 °C for 45 min (air flow rate into the furnace was 100 mL/min, which is about 4.5 m^3^ per 1 kg of raw material). The retort was rotated at a speed of 5 RPM. After the gases had been released, the material was kept at this temperature for an additional 30 min (sample code SHA_650_, where SHA—sunflower husk ash, further in the text-ash).

In all experiments, including the preparation of the ash, the heating rate was maintained at 10 °C min^−1^. The furnace temperature was controlled using a PID controller with a temperature stability of ±1 °C throughout the entire thermal treatment.

The initial mass of sunflower husks used in individual experiments was 3.844, 3.972, 3.708, 4.185, 4.101, and 4.248 kg for samples SHC300, SHC400, SHC500, SHC600, SHC700, and SHC800, respectively. The gravimetric yield of the coals gradually decreased with increasing carbonization temperature, reaching 54.27%, 33.76%, 30.12%, 28.84%, 27.36%, and 26.98% for the SHC300, SHC400, SHC500, SHC600, SHC700, and SHC800 samples, respectively. The observed decrease in the solid yield with increasing temperature is consistent with increased degassing and gradual decomposition of hemicellulose, cellulose, and lignin, resulting in increased volatilization and enrichment of the residual carbon matrix. When the SHA650 sample was prepared, the initial mass of sunflower husks was 3.534 kg, and the gravimetric ash yield was 4.12%.

The temperature range of 300–800 °C chosen in this study was deliberately selected to cover the entire spectrum of primary and secondary stages of biomass decomposition, from the initial degradation of hemicellulose to the advanced carbon structuring associated with the aromatic condensation of lignin. It is well known that hemicellulose decomposes predominantly in the range of 200–350 °C, cellulose decomposes in the range of 300–400 °C, while lignin demonstrates a much wider decomposition range extending up to 800 °C, which significantly contributes to coal aromatization and structural stabilization [56].

At temperatures around 300–400 °C, primary degassing prevails, resulting in a high release of oxygen-containing volatiles and a relatively high yield of coke residue. Increasing the temperature to 500–600 °C shifts the reaction mode towards secondary cracking and gradual carbon enrichment, while above 700 °C, condensation and graphitization processes become dominant, leading to the formation of smaller but more carbon-dense solid residues [57]. Similar temperature-dependent changes in the structure of char have been observed in recent studies of thermochemical conversion of lignocellulose raw materials [58].

It is important to note that the chosen upper limit of 800 °C allows for the transition to highly carbonized semi-graphite structures, which are particularly relevant for adsorption and catalytic applications. This is consistent with recent studies showing that elevated pyrolysis temperatures enhance aromatic condensation and reduce the number of oxygen-containing functional groups on the surface [59].

Another key aspect of this study is the consideration of different reaction atmospheres. Although traditional pyrolysis is typically conducted in inert gases such as N_2_ or Ar, recent studies have shown that the choice of atmosphere significantly affects the distribution of products, secondary reactions, and mineral transformations. For example, pyrolysis using CO_2_ can promote gasification reactions and alter the reactivity of coal, while N_2_ and Ar provide relatively inert conditions that favor the preservation of carbon matrix structure [60]. Additionally, comparative studies have shown that even subtle differences in the composition of the inert gas can influence pore development and the kinetics of volatile matter release [61].

The morphology and structure of the sunflower seed husk char were analyzed using a S-4800 scanning electron microscope (Hitachi, Tokyo, Japan). The structural analysis of the samples was performed using an EDX Quantox-200 fluorescence spectrometer (Bruker, Ettlingen, Germany) [62].

Samples of the obtained chars (5 g) were placed in glass beakers, 100 mL of distilled water was added, and the mixture was stirred for 1 h. The suspension was filtered, and the pH of the solution was determined in the filtrate using a pH 211 pH meter (Hanna, Vöhringen, Germany). The study was conducted on three samples of each material.

The samples of chars were studied using infrared spectroscopy. The registration and processing of IR spectra was carried out on the VER-TEX 70 IR Fourier spectrometer (Bruker Optik GmbH, Ettlingen, Germany) in the frequency range from 4000 to 500 cm^−1^ and using the PIKE MIRacle ATR (PIKE Technologies, Madison WI, USA) single-pass total internal reflection (STIR) attachment with a germanium crystal. The results were processed using the OPUS 7.2.139.1294 software (University of Stuttgart, Stuttgart, Germany).

The surface area was determined based on the Brunauer, Emmett, and Teller theory of gas adsorption using multi-point measurements on a NOVA 2200 instrument (Quantachrome Corp., Boynton Beach, FL, USA) [63]. This method is used to determine the surface area of deposited particles with specific external surfaces ranging from 1 to 2000 m^2^/g. The error of this method is no more than 5%.

X-ray phase analysis is based on obtaining data on the chemical composition of a sample using powder X-ray diffraction. The main objective of X-ray phase analysis is to identify different phases in a mixture based on the analysis of the diffraction pattern produced by the sample under study. The determination of a substance in a mixture is based on the set of its interplanar distances and the relative intensities of the corresponding lines on the X-ray diffraction pattern [64]. The X-ray diffraction patterns of the samples were recorded using a Bruker D8 Advance diffractometer (Bruker, Germany) under the following conditions: step size of 0.10 with a step delay of 2 s; sampling frequency of 5; measurement range from 5 to 80 2θ, and their interpretation was performed using the Profex software v. 5.6.1 (Nicola Döbelin, Bettlach, Switzerland) with the Crystallography Open Database (COD) database.

## 3. Results and Discussion

**Figure 3:**
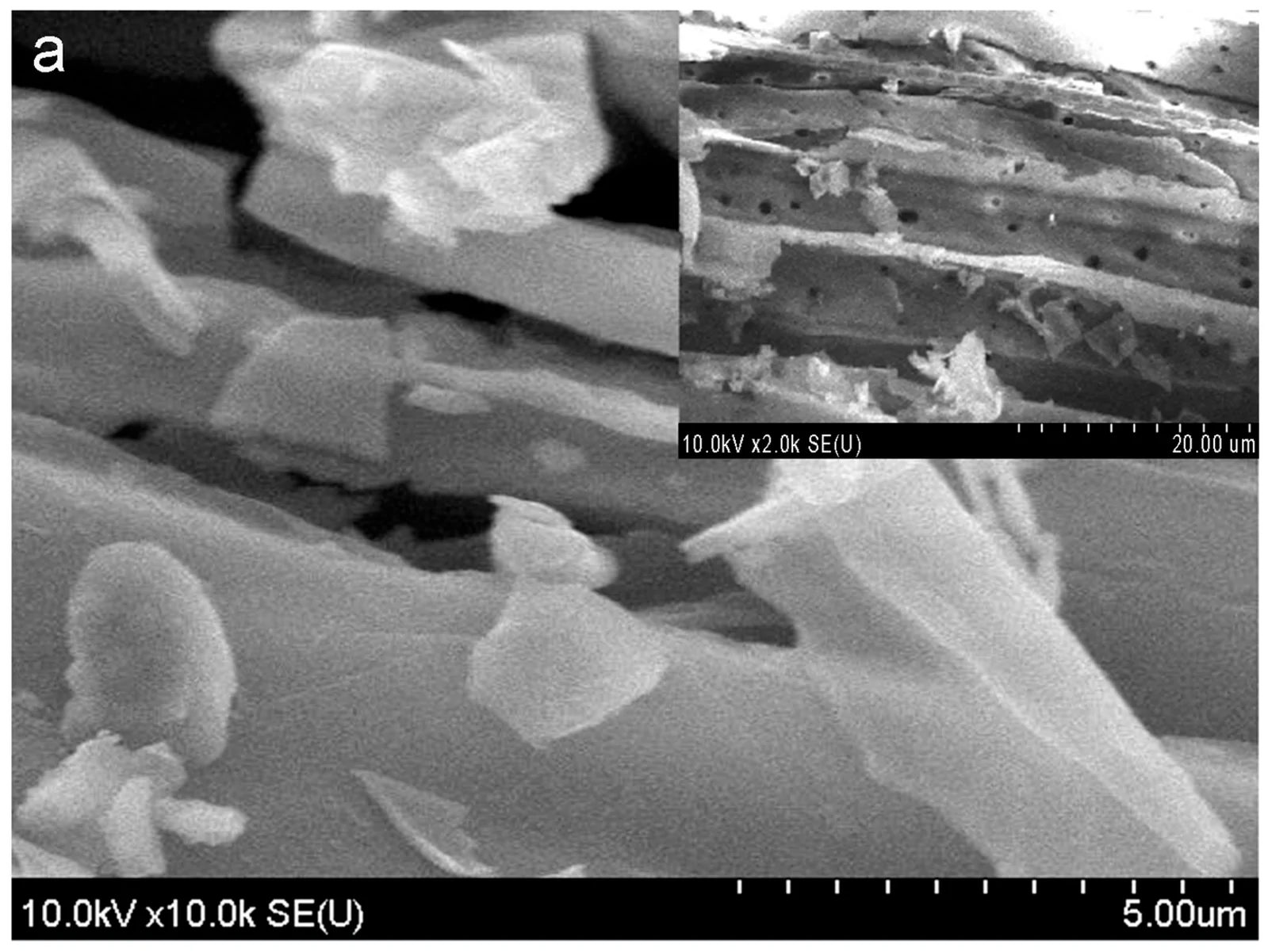

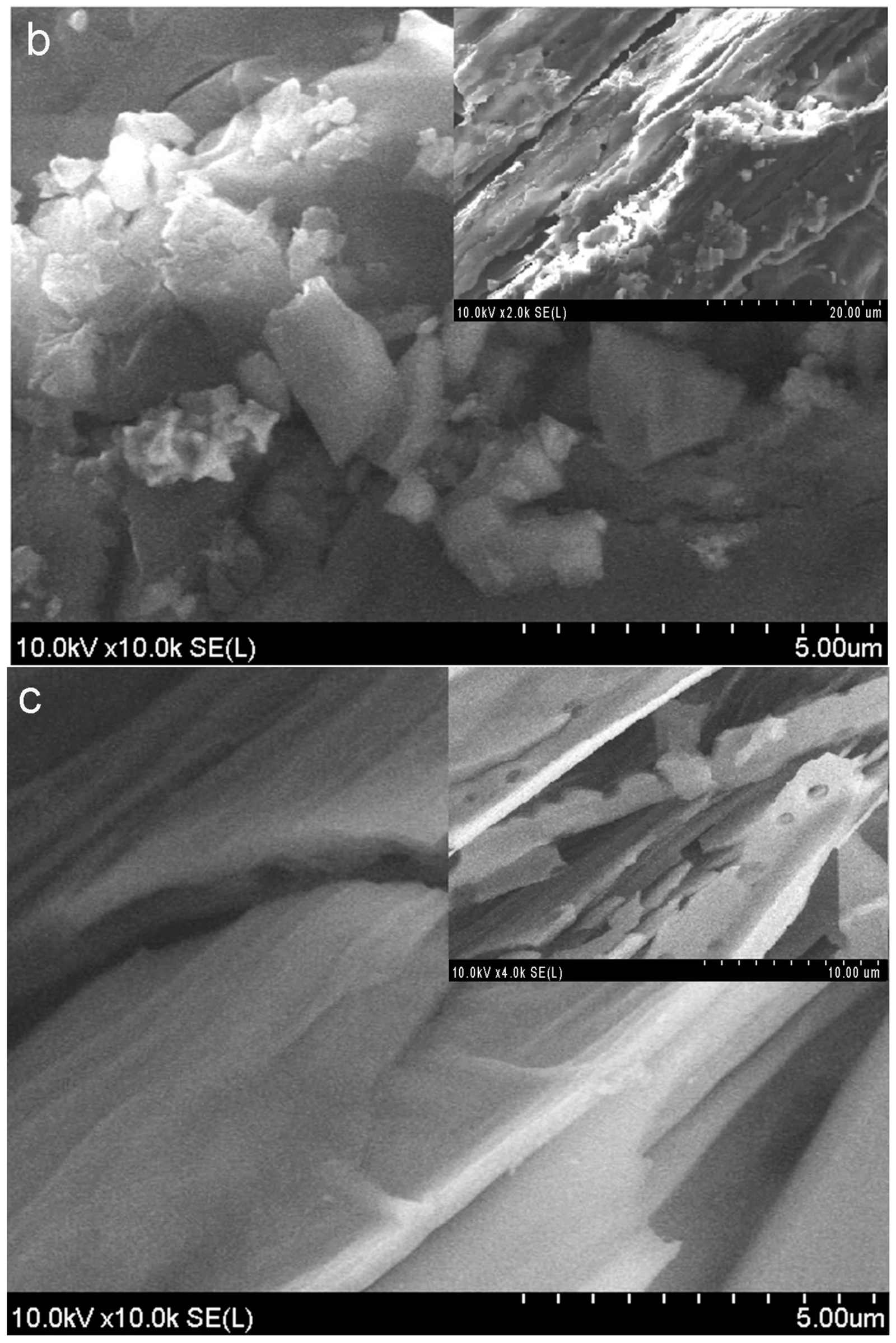

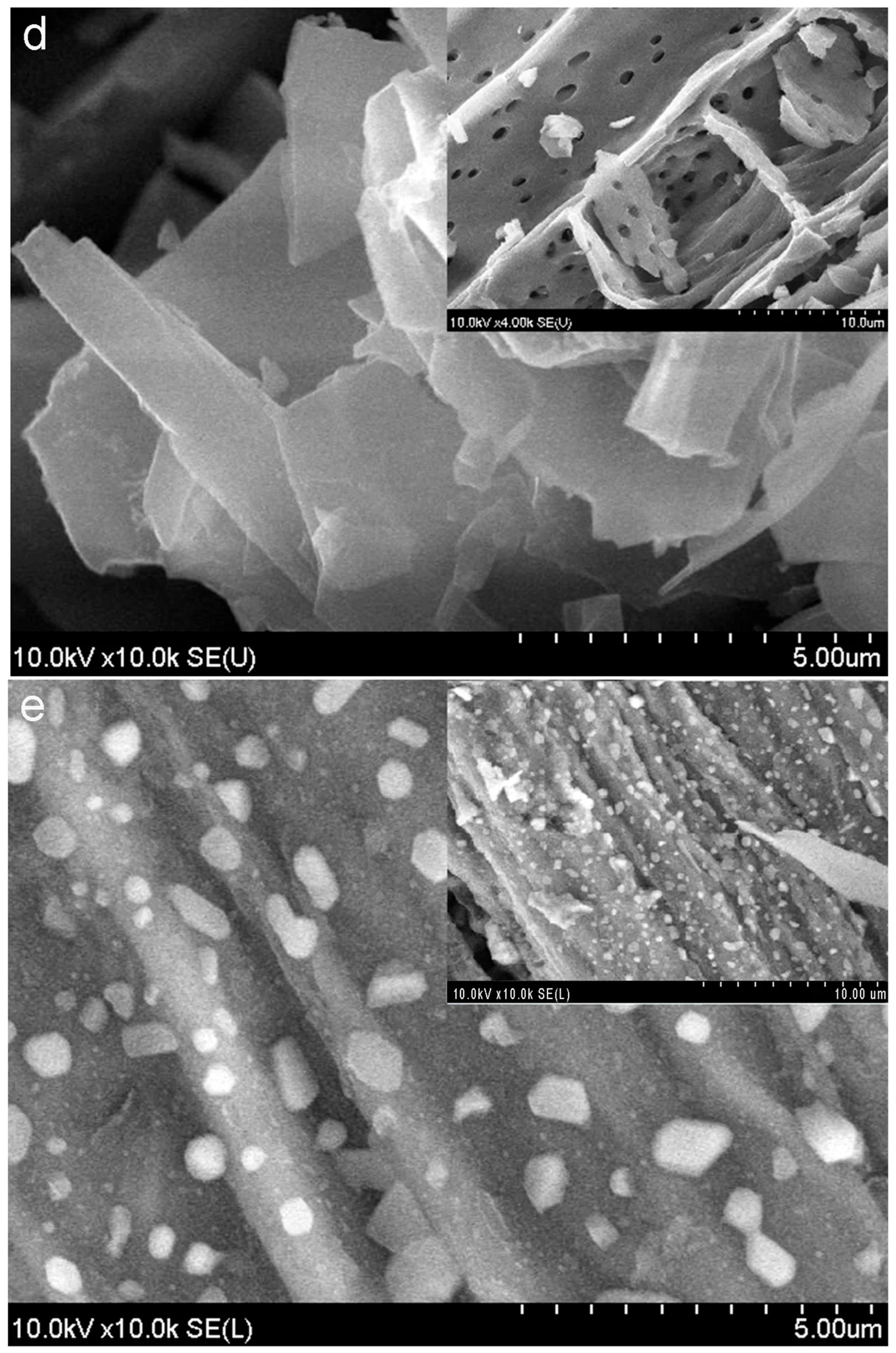

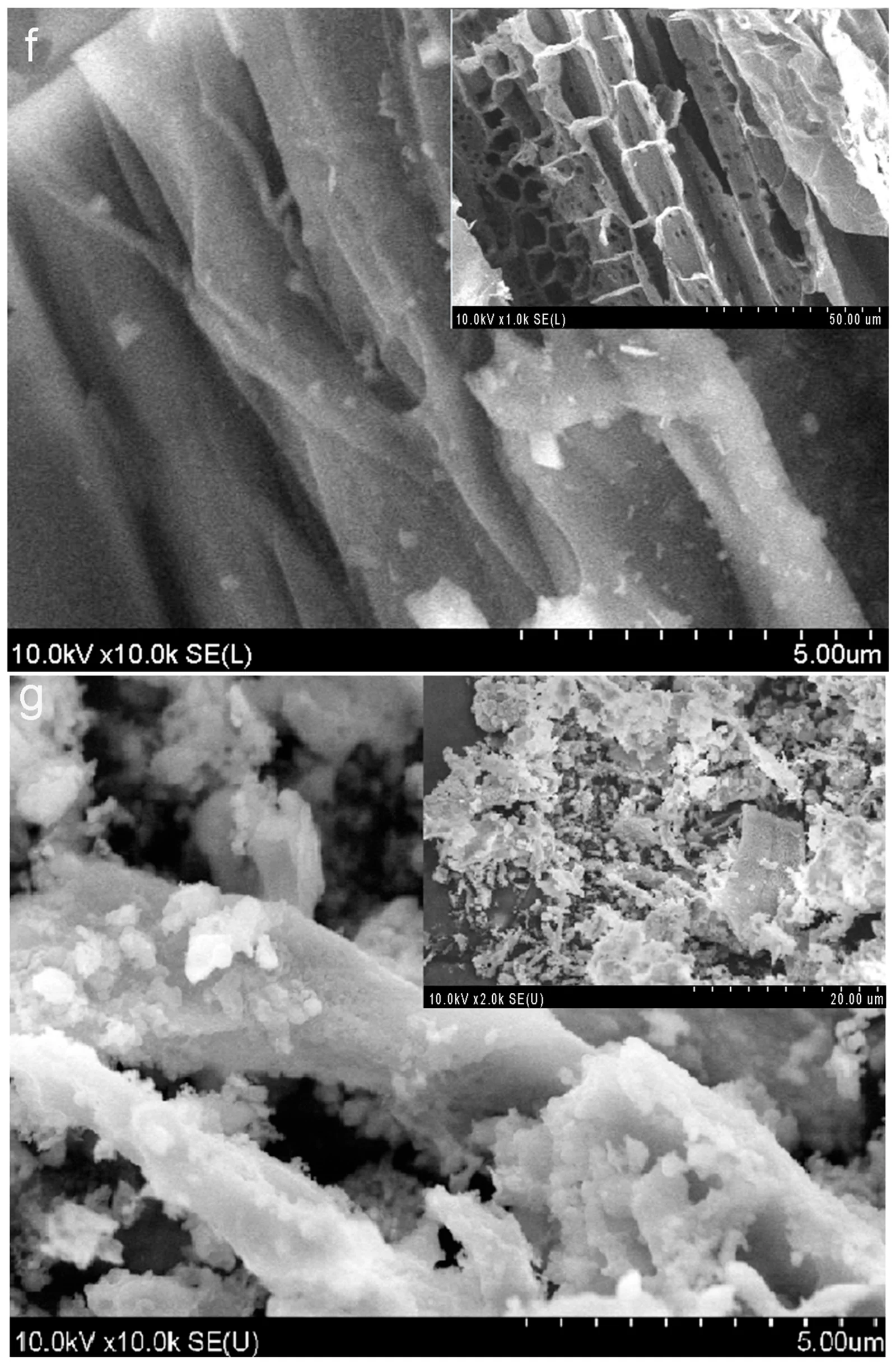
SEM images of the microstructure of sunflower seed husks obtained at different processing temperatures and carbonization environments. (**a**)—SHC_300_; (**b**)—SHC_400_; (**c**)—SHC_500_; (**d**)—SHC_600_; (**e**)—SHC_700_; (**f**)—SHC_800_; (**g**)—SHA_650_.

Figure 3 shows the morphological changes in sunflower husk depending on the temperature and carbonization environment.

The visual appearance of the chars obtained in an inert environment remained consistently black at all the studied processing temperatures, while the material obtained in an oxidizing environment had a light gray color due to complete oxidation and removal of carbon. All the presented micrographs show a general pattern of changes in the structure of the husk with increasing temperature, which includes the gradual volatilization of the main components of the plant raw material (lignin, cellulose, and hemicellulose), the reduction in the thickness of the cell walls, the increase in the porosity of the material, and the densification of the carbon framework [65,66,67].

At a pyrolysis temperature of <400 °C, the structure of the char is still similar to that of the untreated husk. Due to the incomplete removal of volatile substances, the surface area of the material does not actually change. At this temperature, only the initial destruction of hemicellulose occurs, and the majority of lignin and cellulose have not yet begun the process of carbonization [68,69,70]. As the temperature increases to 400–500 °C, the destruction of hemicellulose is completed, the main stage of gas removal from cellulose is completed, and the main carbonization of lignin begins, resulting in a significant increase in the carbon content [71,72]. At high-temperature pyrolysis (600–800 °C), there is a significant change in the morphology of the material, with a clear appearance of a cellular structure and an increase in porosity [33,73], which is crucial for its use in elastomeric compositions [74,75,76,77]. It is worth noting that the surface of the sunflower char has evenly distributed light inclusions, which are likely to be the ash mineral residues characteristic of sunflower husk (silica, calcium, and potassium oxides) [24,78]. At 800 °C, the process of lignin carbonization is complete, and the material exhibits maximum thermal stability and hydrophobicity.

In the case of husk carbonization in the presence of oxygen (SHA_650_), there is a significant change in the structure of the carbonized product. The presence of oxygen at high temperatures contributes to the combustion of the carbon skeleton and the oxidation of the mineral components [79], resulting in the formation of defects and a loose structure characteristic of ash residues [80].

Based on the material structure, the most promising carbonized products for use in elastomeric compositions are those obtained at temperatures of 600–800 °C in an inert environment, as they retain a fibrous structure and a well-developed porosity, which can improve the interaction with polymer chains and affect the strength properties of the final products. Zhang et al. [81] have noted the impact of the porous structure of char on the physical and mechanical properties of biocomposites. They have determined that increasing the pyrolysis temperature reduces the number of polar functional groups in char, which in turn increases its affinity for non-polar polymer groups. Additionally, it has been observed that the high porosity of char has a significant effect on the physical and mechanical properties of the biocomposite. Similar findings have been reported by Nizamuddin et al. [82] and Zhang et al. [83]. Low-temperature pyrolysis products (˂500 °C) may contain excess volatile substances that can be released during the vulcanization process of rubber mixtures, resulting in defects in the final material. In the case of husk pyrolysis with oxygen access, the sample lacks the carbon skeleton necessary for strengthening the elastomer.

Carbonized biomass from various plant materials is a high-carbon substance obtained through the thermal decomposition of plant raw materials [84]. In addition to carbon, carbonized biomass may contain nitrogen, calcium, potassium, sulfur, and other elements [85]. The elemental composition of sunflower husk carbonized biomass obtained at different processing temperatures is shown in Table 1. The structural analysis of the samples was performed using an EDX Quantox-200 fluorescence spectrometer (Bruker, Germany).

The data showed that with an increase in the carbonization temperature, the carbon content increases (up to 600 °C) and the oxygen content decreases, which is explained by the fact that the organic components of sunflower husks were continuously decomposed and carbonized with an increase in the processing temperature, resulting in an increase in the carbon content [77,86,87,88]. This trend is consistent with the results of previous studies [89,90,91,92]. When the pyrolysis temperature exceeded 600 °C, the carbon content in the sunflower husk char decreased, which is due to the destruction of the vesicular structure of the chars at higher temperatures [81,93]. The changes in the sulfur, silicon, and phosphorus content can be considered insignificant, similar to the results obtained by Mimmo et al. [94]. The increase in the concentration of alkali metals during heating from 300 to 800 °C is due to the volatilization of the organic matter in the form of gases, water, and resins. The total mass of the char decreases, while the amount of metals remains the same as in the original sunflower husks [24]. When sunflower seed husks are processed in an oxidizing environment, the organic matter is almost completely oxidized, resulting in a mineral fraction enriched with potassium and calcium carbonates.

**Figure 4:**
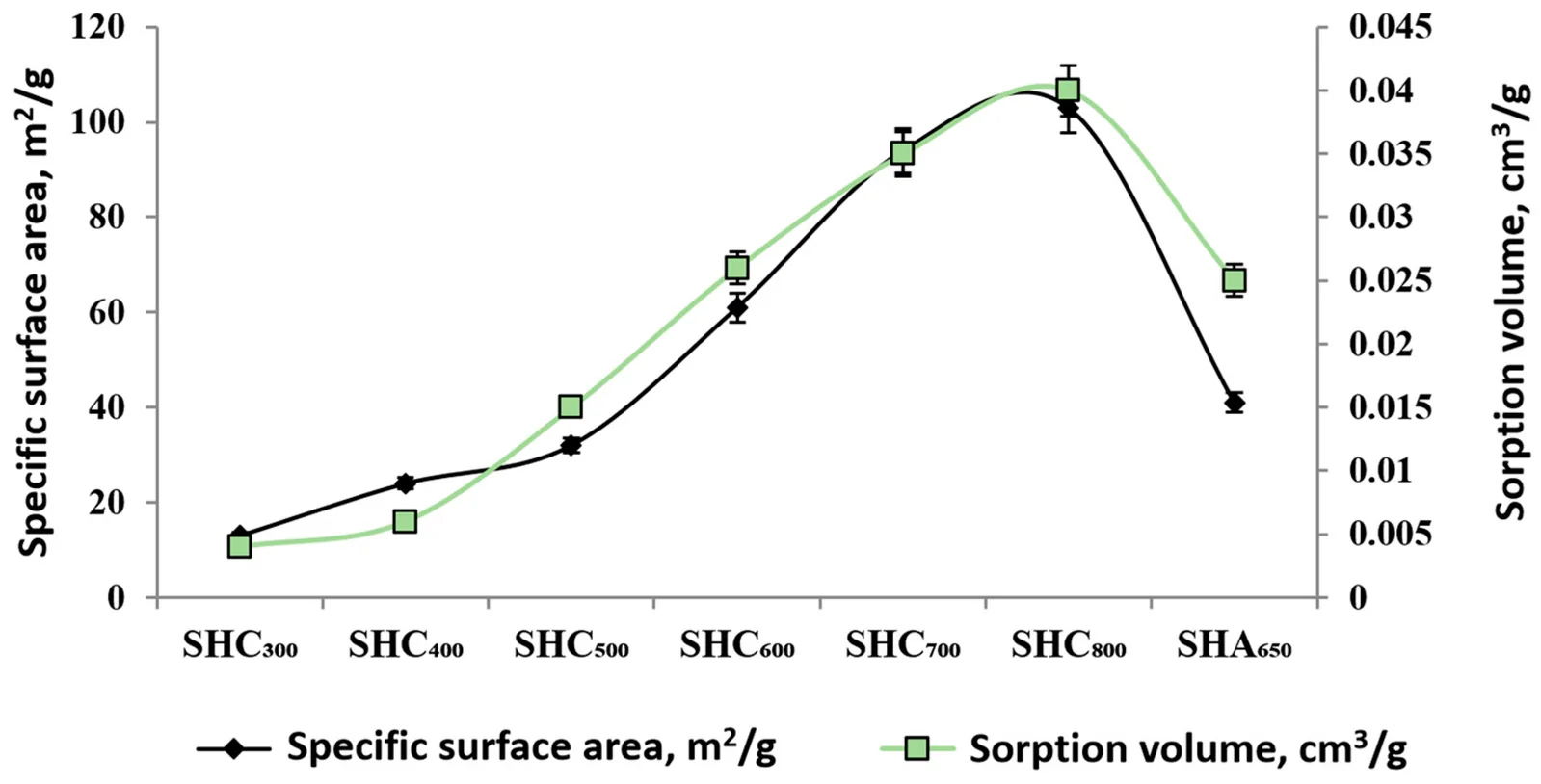
Structural properties of the studied materials.

The quality of the char and its physical and chemical characteristics depend on the properties of the raw materials used, as well as the conditions of thermal processing. The processing temperature has a significant impact, as it affects the surface area of the char and its sorption properties [25,36,49,50]. The changes in the specific surface area and sorption volume of the studied chars, depending on the temperature and processing environment, are shown in Figure 4.

**Figure 5:**
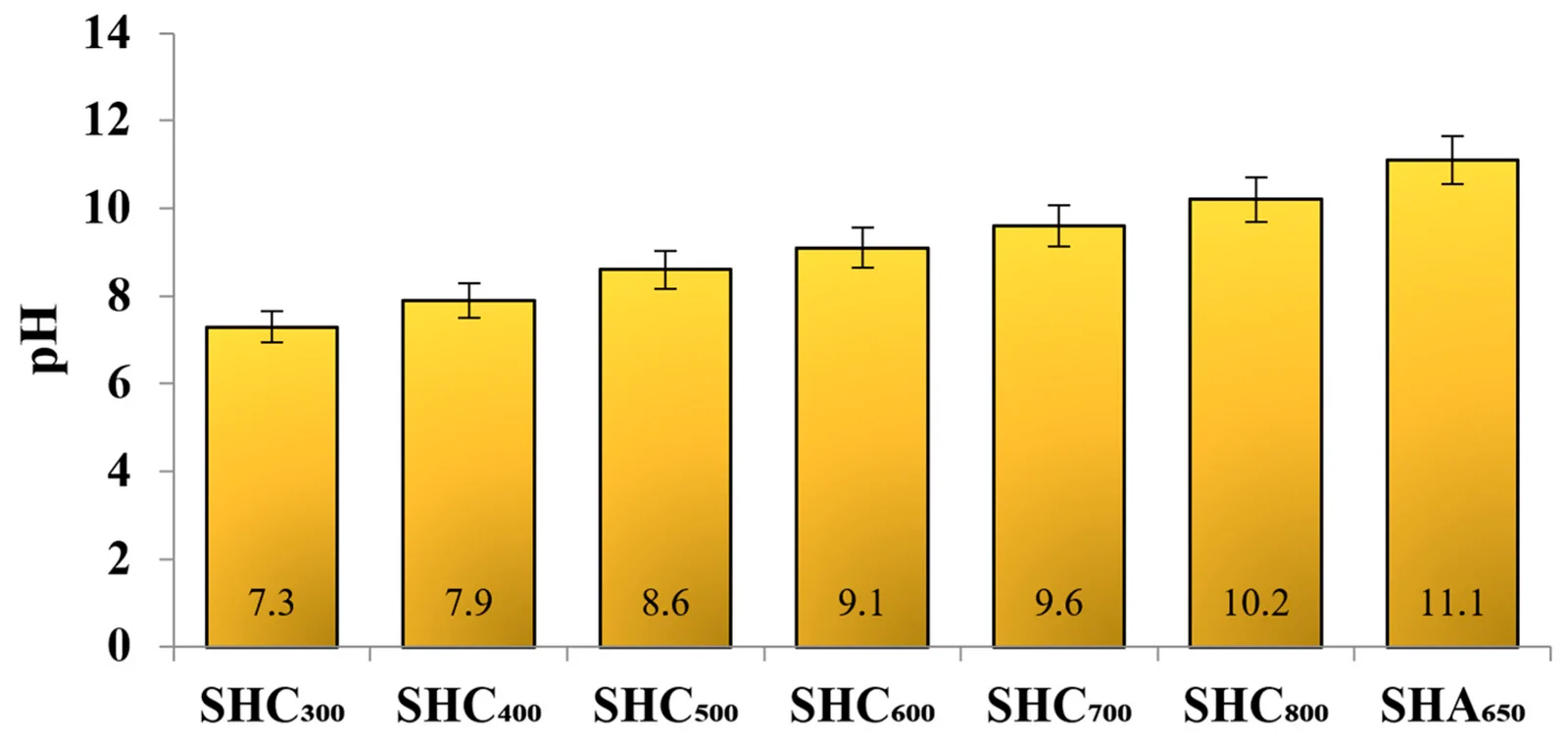
pH of the aqueous suspension of the studied materials.

The specific surface area and sorption volume of the sunflower husk char increase from 13 ± 0.65 m^2^/g to 103 ± 5.2 m^2^/g with an increase in the carbonization temperature, which is due to the fact that at low processing temperatures, the organic components (hemicellulose, cellulose, and lignin) of the studied material are not completely decomposed, resulting in a smaller number of pores on the surface of the char. However, as the temperature increases, the organic substances, as well as the resins and oils, in the biomass are decomposed, forming a highly porous structure [77,95,96,97,98]. Among all the temperature treatments studied for sunflower husk, carbonization at 800 °C proved to be the most effective, as the specific surface area of the resulting material was 103 m^2^/g. As a result of increasing the carbonization temperature, the specific surface area increased by a factor of 7.9. There are numerous studies in the literature that focus on the effect of processing temperature on the various physical and chemical properties of biomaterials, including their specific surface area [28,29,32,41,42,43]. For example, Hung et al. [41] found that increasing the pyrolysis temperature of biomaterial from 700 to 800 °C significantly increased the surface area from 6 to 108 m^2^/g. However, Wystalska et al. [25] did not observe any significant change in the specific surface area of sunflower husk pellets with temperature variations. Nevertheless, the maximum specific surface area of sunflower seed husk char obtained through carbonization in an inert argon environment at 800 °C may not be sufficient for enhancing the performance of elastomeric compositions. To further increase the specific surface area, it is necessary to employ additional CO_2_ activation processes [99]. This was confirmed by a study by Januszewicz et al. [100], which studied char obtained from the waste of various plant crops and subjected it to an activation process at a temperature of 800 °C and a constant CO_2_ flow rate, resulting in an increase in the specific surface area (from 4 to 10 times) to 725 m^2^/g for corn cobs, 534.9 m^2^/g for pistachio shells, 523 m^2^/g for coconut shells, and 393 m^2^/g for walnut shells (Figure 5).

The pH values (the error in all measurements did not exceed 5%) of the obtained char samples gradually increase with increasing temperature (from 7.3 to 11.1), which is associated with a decrease in the content of acidic functional groups [101] and an increase in the proportion of alkaline substances, hydroxide and carbonate mineral phases, in the chars at high temperatures, which can increase the pH [42,102,103,104]. The increase in pH values with increasing temperature is consistent with the data obtained in [101,105,106,107]. The predominance of an alkaline environment should be taken into account when formulating elastomeric compositions, as the alkaline nature of the ingredient can significantly affect the vulcanization kinetics of the rubber mixture, acting as an activator, accelerator, or inhibitor of the process, depending on the specific system.

**Figure 6:**
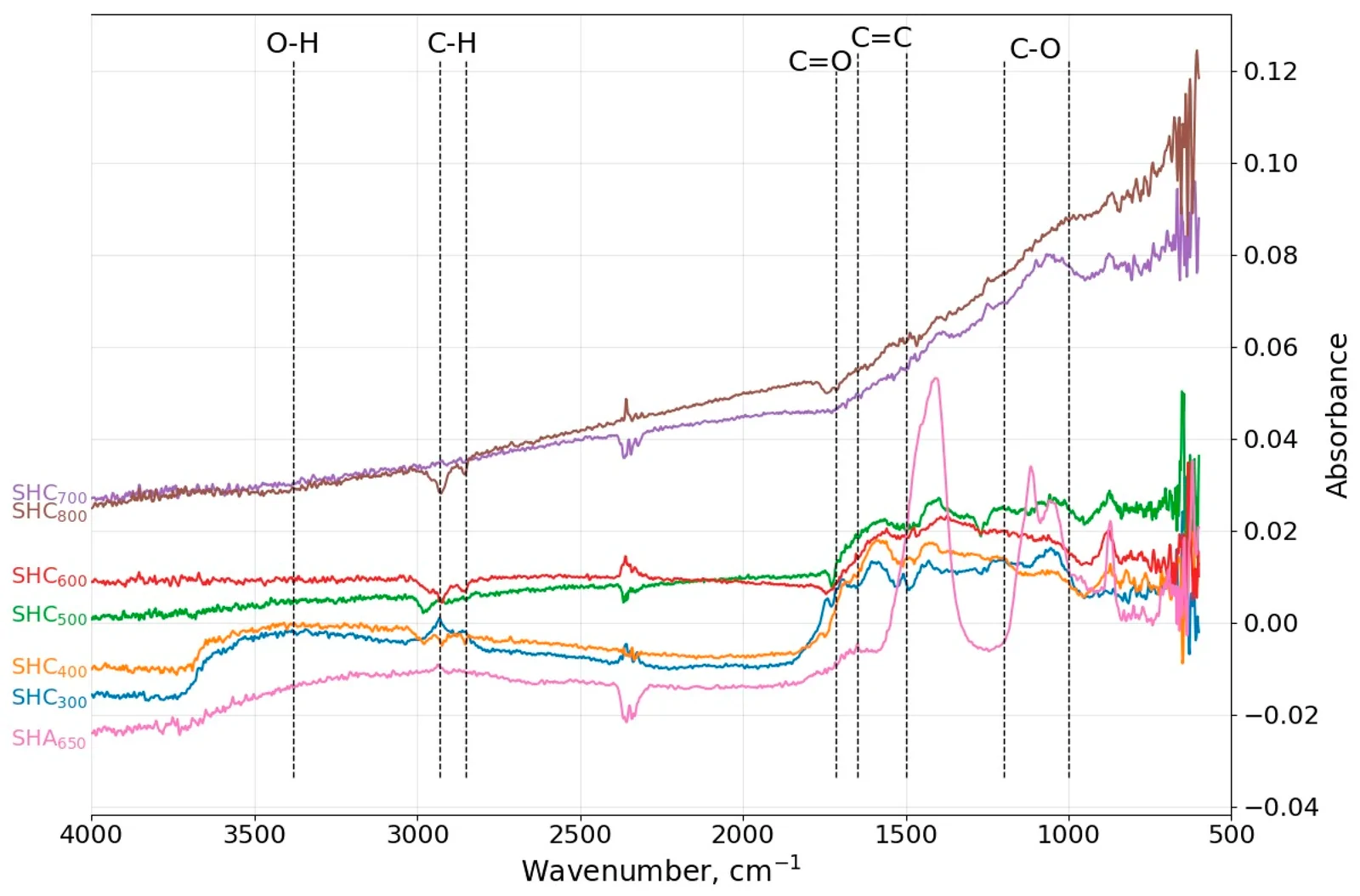
IR spectra of the studied materials.

To determine the types of functional groups present on the surface of sunflower seed husks, depending on the temperature and carbonization environment, an analysis of IR spectra was conducted. The IR spectra of the samples in the 4000–400 cm^−1^ region are presented in Figure 6.

FTIR analysis was used to assess the structural changes in sunflower hull chars obtained at temperatures of 300–800 °C. The spectra demonstrate a gradual transformation of the initial lignocellulose structure into a more aromatic carbon material as the processing temperature increases.

The char obtained at 300 °C is characterized by the presence of intense bands around 3380 cm^−1^, corresponding to the valence vibrations of O–H groups, bands at 2930–2850 cm^−1^ associated with aliphatic C–H bonds, and bands in the regions of 1730–1700 and 1200–1000 cm^−1^, corresponding to C=O and C–O functional groups. This indicates the preservation of a significant portion of the oxygen-containing structures of the initial biomass and incomplete carbonization of the material.

An increase in temperature to 400–500 °C leads to a decrease in the intensity of the O–H, C–H, and C–O bands and an increase in the band around 1600 cm^−1^ associated with aromatic C=C structures. The observed changes indicate the occurrence of dehydration, removal of oxygen-containing groups, and the formation of a more condensed carbon structure, which corresponds to the literature data on the thermal transformation of lignocellulose materials. At temperatures of 600–700 °C, there is a further decrease in the intensity of the O–H and C–O bands, and the 1650–1500 cm^−1^ region, which is associated with the aromatic sp^2^ carbon framework, becomes dominant. This indicates the deepening of deoxygenation processes and the development of a more stable carbon matrix [108].

The maximum degree of structural rearrangement is observed for the sample obtained at 800 °C: oxygen-containing functional groups almost disappear, and the spectrum is predominantly determined by aromatic carbon structures. The weak intensity of bands around 2900 cm^−1^ indicates the presence of only residual aliphatic fragments.

Thus, an increase in the carbonization temperature from 300 to 800 °C is accompanied by a consistent removal of oxygen-containing groups, a decrease in the proportion of aliphatic structures, and an increase in the degree of aromatization of the carbon material. This trend corresponds to the typical mechanisms of thermal conversion of biomass, which involve the destruction of unstable oxygen-containing components in the early stages and the subsequent condensation of aromatic structures at high temperatures [109].

From the point of view of application as a carbon filler for rubber engineering materials, the most promising are chars obtained at 600–800 °C, since they are characterized by a developed aromatic structure, high thermal stability and a smaller number of polar oxygen-containing groups. Samples obtained at 300–400 °C retain a significant part of the original biomass structure, which may limit their compatibility with a hydrophobic polymer matrix. Similar properties of high-temperature bio-coals as alternative carbon fillers have been described for various plant wastes [23,74].

In general, FTIR analysis confirms that the carbonization of sunflower husks at 600–800 °C leads to the formation of the most stable carbon material with characteristics that are promising for use in rubber products.

The IR spectra of the samples obtained during the carbonization of sunflower husks in an argon environment at various processing temperatures demonstrate the preservation of a significant number of oxygen and aliphatic functional groups even at high temperatures. In all the studied spectra, there is a broad band of valence vibrations of the –OH groups in the region of 3700–3200 cm^−1^, and the vibrations of the C-H groups in the region of 3000–2800 cm^−1^ have also been identified [110,111,112,113,114,115]. As the processing temperature of the plant material increases, the peaks in the region of 2900 cm^−1^ should decrease due to the instability of the OH groups at elevated temperatures [107], but in our case, the peak remains pronounced, which may indicate that the aliphatic structures are preserved under these carbonization conditions. Additionally, all the studied spectra show the presence of bands of carbonyl groups C=O (region of 1750–1680 cm^−1^), which decrease with increasing temperature, indicating the active occurrence of decarboxylation processes [107,116,117,118,119,120]. This region demonstrates the most pronounced temperature change. In the case of the region of 1650–1450 cm^−1^, which is responsible for the vibrations of the C=C aromatic rings [121,122,123,124], the opposite trend is observed. At a carbonization temperature of 300 °C for sunflower husks, this band is weak, but at 800 °C, it becomes dominant in the spectrum, indicating the active aromatization of the material. However, in a study [107], as the pyrolysis temperature increased from 400 to 700 °C for beech wood chips, walnut shells, and wheat straw, there was a decrease in the intensity of the C=C bond valence vibration signals, which was attributed to the fact that high temperatures can lead to the breaking of double bonds. The C-O bond valence vibrations were observed in the region of 1100–1050 cm^−1^. This compound is not only a testament to the lignocellulose structure of the material under study [125,126], but it may also indicate the formation of aromatic esters through the integration of oxygen atoms into cyclic carbon structures [127]. It is also worth noting that the region 950–700 cm^−1^ (deformational vibrations of aromatic C-H groups) is preserved, which also indicates an increase in the degree of carbon matrix aromatization [128,129,130].

The IR spectrum of the sample obtained by carbonization in an oxidizing environment differs significantly from the materials obtained in an inert environment. There is a complete disappearance of the C-H (3000–2800 cm^−1^) and C=O (1750–1680 cm^−1^) bands. However, the O-H group valence band remains in the range of 3700–3200 cm^−1^. A characteristic feature of this spectrum is the band in the range of 1450–1410 cm^−1^, which corresponds to the asymmetric valence vibrations of the carbonate ion CO_3_^−2^ [131], and the band in the range of 1150–1000 cm^−1^, which corresponds to the orthophosphate ion PO_4_^3−^ [132]. This is also confirmed by the bands in the range of 880–870 cm^−1^ and 712 cm^−1^, which correspond to the carbonates of alkaline and alkaline-earth metals, primarily CaO and MgO [132], which is consistent with the chemical composition of the material.

Based on the obtained data, it can be concluded that the thermal treatment environment has a significant impact on the chemical and phase composition of the materials. Carbonization of sunflower seed husks in an argon environment produces a carbon-containing material with a well-developed aromatic structure and residual oxygen-containing and aliphatic groups. In contrast, when the tests are conducted in an oxidizing environment, the organic part is completely oxidized, resulting in a mineral part enriched with potassium and calcium carbonates. These findings demonstrate the potential for the controlled production of either carbon-containing materials or ash by varying the thermal treatment environment.

**Figure 7:**
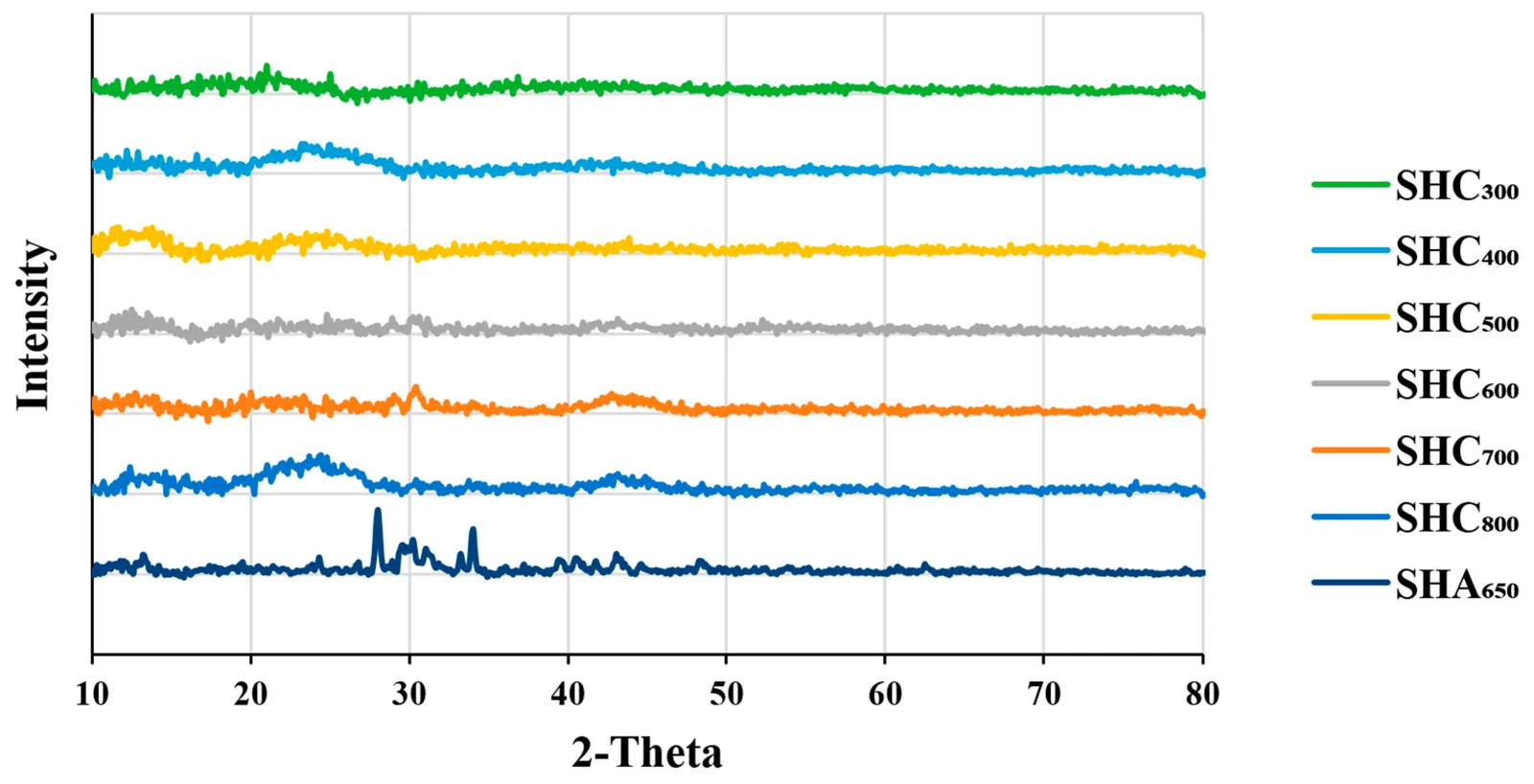
Combined diffraction pattern of sunflower husk chars obtained at different processing temperatures.

X-ray diffraction allows for the analysis of the mineral components present in the studied materials. Figure 7 shows the diffraction patterns of the chars obtained at various temperatures (300, 400, 500, 600, 700, and 800 °C without oxygen access and at 650 °C in an oxidizing environment) during carbonization.

**Figure 8:**
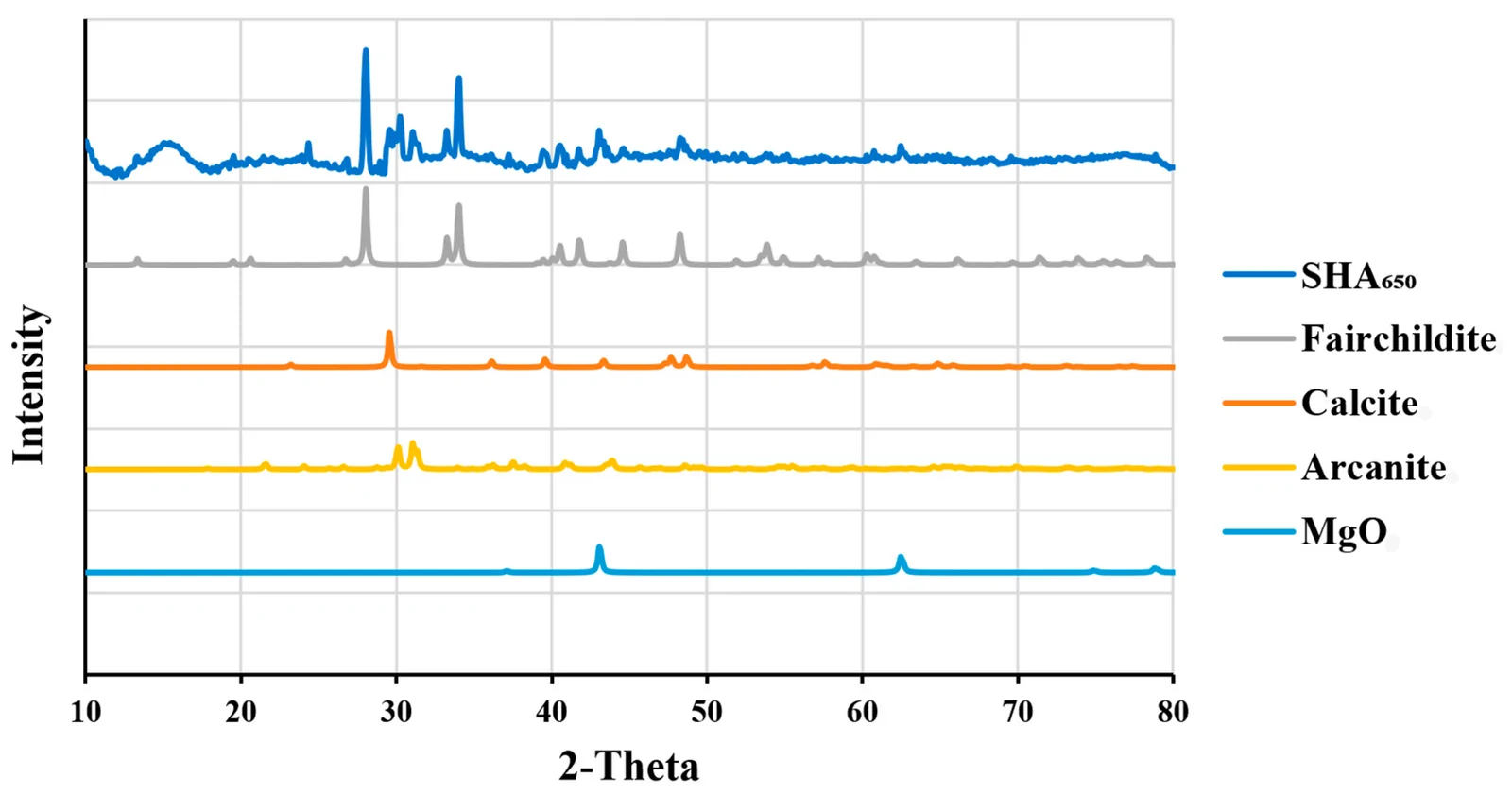
Diffractogram of ash in comparison with reference diffractograms.

A joint analysis of XRD and EDX data showed that as the temperature of sunflower husk carbonization increases from 300 to 800 °C, there is a consistent decrease in oxygen content and a relative enrichment of chars with carbon and mineral elements, primarily K, Ca, and Mg. This is manifested in the diffraction patterns by a transition from the predominantly diffuse profile of low-temperature samples to more pronounced broad maxima in the 2θ = 21–25° and 42–44° regions, which are characteristic of the weakly ordered turbostratic carbon obtained from lignocellulosic biomass [133]. The amorphous nature of the carbon is due to the partial carbonization of lignin, hemicellulose, and cellulose structures during the carbonization process at elevated temperatures, and under these conditions, there is no complete transformation into crystalline graphite [134,135]. Even as the mineral content increases, the distinct reflections of crystalline phases in the chars remain weak, indicating their low degree of crystallinity, low dispersion, or overlapping with the broad background of the carbon matrix. Thus, the main effect of increasing the carbonization temperature is the development of the carbon structure and the concentration of the mineral portion, rather than the formation of a large number of well-crystallized phases [53]. In contrast, the ash obtained at 650 °C in an air atmosphere exhibits the formation of a distinctly crystalline mineral phase (Figure 8).

Given the high content of K, Ca, Mg, S, and P, and the low content of Cl and Si, it is most likely that kalium and calcium-containing carbonate phases, including calcite and mixed K–Ca carbonates such as fairchildite, as well as kalium sulfate phases such as arcanite, are present. The presence of Mg indicates the possible presence of magnesium oxide, carbonate, or mixed phases. K/Ca/Mg phosphate phases are also not excluded. In contrast, the presence of sylvite (KCl) and quartz (SiO_2_) as the main phases is unlikely, as the Cl and Si content is low according to EDX data [132,136].

## 4. Conclusions

Thermochemical treatment (carbonization in the temperature range of 300–800 °C in various environments) of biodegradable plant-based waste leads to the formation of materials with different physical and chemical surface characteristics. In this study, the changes in the properties of chars were investigated depending on the temperature and processing environment. It was found that the carbon content in the chars increases with increasing temperature during carbonization in an inert environment, while the carbon content is minimal in the case of processing in an oxidative environment. It was also determined that the pH value increases with increasing temperature and during processing in an air environment. Another parameter that showed a tendency to increase with increasing temperature during processing in an inert environment was the specific surface area and sorption volume (with the exception of the sample obtained at a temperature of 650 °C in an oxidative environment). Increasing the carbonization temperature in an inert environment also led to a decrease in the number and intensity of C=O carbonyl functional groups on the surface of the studied chars. In the case of heat treatment of sunflower seed husks in an oxidative environment, oxidation of the organic part of the raw material and the formation of a mineral fraction were observed.

A combined analysis of X-ray diffraction and EDX data showed that as the temperature of sunflower seed husk carbonization increases from 300 to 800 °C in an inert environment, there is a consistent decrease in the oxygen content and a relative enrichment of the chars with amorphous carbon and mineral elements, primarily K, Ca, and Mg. The ash showed distinct crystalline mineral phases.

The alkaline nature of all the studied chars, as well as the presence of sufficient amounts of metals, makes these materials promising for research as functional additives (co-vulcanizing or secondarily activating agents) for elastomeric compositions, as it is likely that the addition of chars to the compositions will contribute to the additional creation of a valid vulcanization agent, which in turn will affect the kinetic parameters of rubber compound processing. At the same time, the high content of amorphous carbon, the presence of oxygen-containing functional groups, and the well-developed specific surface area of sunflower seed husk char obtained at temperatures of 500–800 °C create potential for using these materials in elastomeric compositions as fillers.

## Figures and Tables

**Table 1:** Elemental composition of sunflower seed husks under different temperature conditions.

Sample	Elements, %							
	C	O	K	Mg	Ca	P	Si	S
SHC_300_	61.99	36.31	0.57	0.35	0.56	0.06	0.06	0.03
SHC_400_	70.21	23.39	3.79	0.88	1.46	0.11	0.07	0.10
SHC_500_	73.48	21.34	2.38	0.59	1.99	0.08	0.05	0.08
SHC_600_	77.89	14.72	4.31	0.66	2.12	0.13	0.07	0.10
SHC_700_	76.00	12.37	5.93	1.18	3.69	0.36	0.26	0.21
SHC_800_	72.74	11.44	8.70	1.64	4.36	0.71	0.11	0.30
SHA_650_	6.07	42.05	26.70	4.53	15.46	1.75	0.63	2.08

## Data Availability

The original contributions presented in this study are included in the article. Further inquiries can be directed to the corresponding author.
